# Application of Liquid Chromatography Coupled to Mass Spectrometry for Direct Estimation of the Total Levels of Adenosine and Its Catabolites in Human Blood

**DOI:** 10.3390/ph17030345

**Published:** 2024-03-07

**Authors:** Jakub Šofranko, Peter Mitro, Zora Lazúrová, Martin Jozef Péč, Tomáš Bolek, Renata Péčová, Matúš Dohál, Matej Samoš, Radovan Murín

**Affiliations:** 1 Department of Medical Biochemistry, Jessenius Faculty of Medicine in Martin, Comenius University in Bratislava, 036 01 Martin, Slovakia; sofranko4@uniba.sk; 2 Cardiology Clinic VUSCH, Šafarik University, 040 11 Košice, Slovakia; 3 Department of Internal Medicine I, Jessenius Faculty of Medicine in Martin, Comenius University in Bratislava, 036 01 Martin, Slovakia; 4 Department of Cardiology, Teaching Hospital in Trenčín, 911 71 Trenčín, Slovakia; 5 Department of Pathological Physiology, Jessenius Faculty of Medicine in Martin, Comenius University in Bratislava, 036 01 Martin, Slovakia; 6 Biomedical Center Martin, Jessenius Faculty of Medicine in Martin, 832 32 Bratislava, Slovakia; 7 Division of Invasive and Interventional Cardiology, Department of Acute and Interventional Cardiology, Mid-Slovakian Institute of Heart and Vessel Diseases (SÚSCCH, a.s.) in Banská Bystrica, 974 01 Banská Bystrica, Slovakia

**Keywords:** adenosine, inosine, xanthine, hypoxanthine, blood, LC–MS

## Abstract

Adenosine is a multifunctional nucleoside with several roles across various levels in organisms. Beyond its intracellular involvement in cellular metabolism, extracellular adenosine potently influences both physiological and pathological processes. In relation to its blood level, adenosine impacts the cardiovascular system, such as heart beat rate and vasodilation. To exploit the adenosine levels in the blood, we employed the liquid chromatography method coupled with mass spectrometry (LC–MS). Immediately after collection, a blood sample mixed with acetonitrile solution that is either enriched with ^13^C-labeled adenosine or a newly generated mixture is transferred into the tubes containing the defined amount of ^13^C-labeled adenosine. The ^13^C-enriched isotopic adenosine is used as an internal standard, allowing for more accurate quantification of adenosine. This novel protocol for LC–MS-based estimation of adenosine delivers a rapid, highly sensitive, and reproducible means for quantitative estimation of total adenosine in blood. The method also allows for quantification of a few catabolites of adenosine, i.e., inosine, hypoxanthine, and xanthine. Our current setup did not allow for the detection or quantifying of uric acid, which is the final product of adenosine catabolism. This advancement provides an analytical tool that has the potential to enhance our understanding of adenosine’s systemic impact and pave the way for further investigations into its intricate regulatory mechanisms.

## 1. Introduction

Adenosine, comprised of adenine and D-ribose, serves vital roles in all living organisms on several levels, from cellular metabolism through physiological regulation to the etiology of several diseases [1,2,3,4,5,6,7,8]. Beyond its structural role in nucleotides forming DNA or RNA molecules, it functions in energy transfer and signal transduction. Its diverse signaling and regulatory roles make adenosine a neurotransmitter and potent vasodilator [5,9,10]. Intravenous administration of a restricted amount of adenosine affects the atrioventricular node to stop arrhythmia, helps to dilate the blood vessels, and influences the blood flow [5,9,11]. Adenosine overdose can lead to adverse effects, including chest pain, faintness, shortness of breath, and sensory tingling. Serious complications encompass worsening dysrhythmias and low blood pressure [12]. Elevated adenosine levels in the blood can function as an immunotoxin, disrupting or destroying immune cells, and as a metabotoxin, causing health issues at sustained high concentrations [13]. Chronic adenosine elevation, a consequence of adenosine deaminase deficiency (OMIM 102700), leads to severe combined immunodeficiency phenotype. Therefore, it can be concluded that the level of adenosine in the blood is a crucial parameter underlying the extent of its effects.

The intricate interplay between adenosine levels in the bloodstream and its role in regulating cardiovascular, neuronal, and immunological functions has become a subject of increasing interest and research. It is hypothesized that understanding the correlation between blood adenosine levels and its physiological responses holds significant promise for unraveling mechanisms that govern heart rate, vasodilation, and other crucial aspects of cardiovascular homeostasis. In this context, advancements in analytical techniques, such as liquid chromatography coupled with mass spectrometry (LC–MS) [14], provide valuable tools for quantifying adenosine levels in blood [15]. This method capitalizes on the synergistic combination of liquid chromatography, which separates individual components in a complex mixture, and mass spectrometry, which identifies and measures these components based on their mass-to-charge ratio. Even though several protocols for LC-based adenosine level estimation in plasma, serum, or blood have already been proposed, they operate without the appropriate internal standard reflecting the putative chemical of enzymatically stimulated changes in adenosine levels during sample processing and analysis. Those methods provide data about the level of adenosine in plasma samples of healthy donors, which vary significantly. Indeed, adenosine remained undetected or varied in ranges from 43 ± 3 nM to 5.6 ± 1.7 μM [15,16,17,18,19,20]. The authors of the recently published method based on the LC–MS/MS method, when quantification of adenosine in the blood is performed by using an external standard, conclude that adenosine levels in the blood are dependent on the time of investigation and may range from 0.33 ± 1.03 to 0.70 ± 1.89 μM, depending on the examination time points [15]. Moreover, it is largely known that adenosine is rapidly generated or cleared in blood, therefore, its estimation is challenging. It is important to stop the process in samples right after collection. For this purpose, the compound erythro-9-amino-β-hexyl-α-methyl-9H-purine-9-ethanol hydrochloride can be used to stabilize adenosine level in plasma [21]. As an alternative, the estimation of total adenosine level in whole blood after protein denaturation and analyte extraction can be considered. For protein precipitation, perchloric acid or organic solvents such as methanol or acetonitrile can be used. This only confirms the need for use of internal standards in adenosine evaluation protocols.

**Scheme 1:**
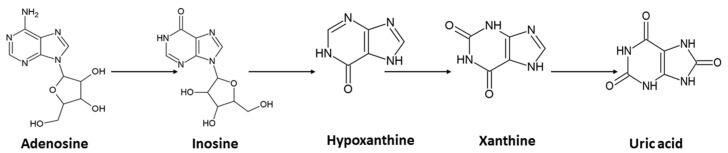
Order of production of adenosine catabolites, inosine, hypoxanthine, xanthine, and uric acid and its chemical structures. Scheme was created by authors using freeware version of ChemSketch software v. 2021.1.0 “https://www.acdlabs.com/ (accessed on 2 September 2022)”.

Adenosine can be generated from several pathways. It can emerge from AMP through catabolism of ATP, or by hydrolyzation of S-adenosylhomocysteine. Following its formation, it can also be further metabolized to inosine, which is subsequently changed to hypoxanthine, xanthine, and, finally, uric acid (Scheme 1.)

With the aim to establish a method that can provide a higher level of standardization, we have exploited the following: (i) the possibility of enriching the blood sample with ^13^C-labeled adenosine before its processing and (ii) the fact that adenosine is a sufficiently polar molecule for its direct injection and subsequent separation and concentration on polar columns before MS detection. In the context of adenosine analysis, we developed an LC–MS assay that is very sensitive, highly specific, and appropriately reproducible. This current method possesses the features of a reliable tool to explore the dynamic variations in adenosine concentrations within the blood. In addition, the versatility and simplicity of the designed LC–MS protocol allow for rapid and simultaneous quantification of adenosine alongside other analytes, such as inosine, hypoxanthine, and xanthine. The method may help to provide data that can contribute to a comprehensive understanding of the roles of blood adenosine in various physiological and pathological states.

## 2. Results and Discussion

**Figure 1:**
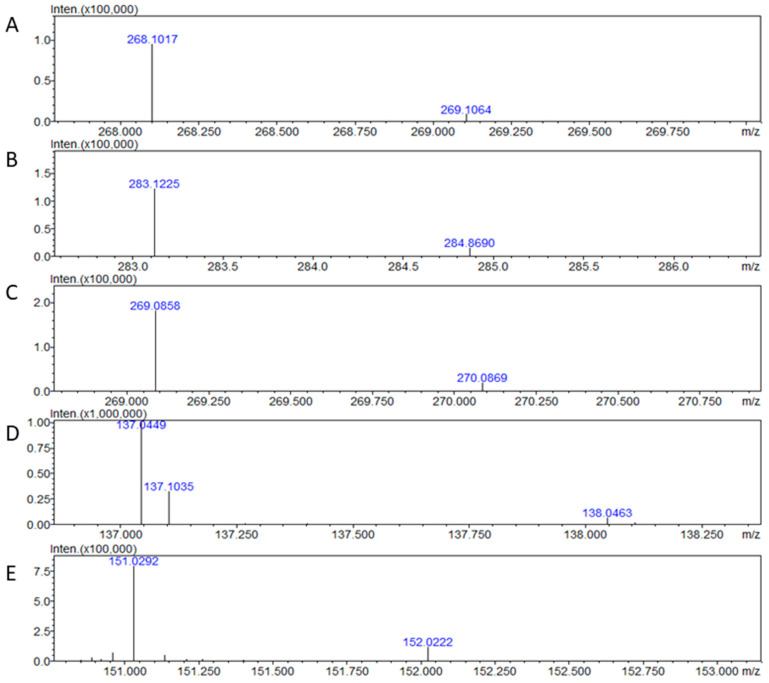
Mass spectra were obtained for adenosine-^12^C_10_,^14^N_5_ (**A**), adenosine-^13^C_10_,^15^N_5_ (**B**), inosine (**C**), hypoxanthine (**D**), and xanthine (**E**). The spectra were obtained after direct injection of 2 ng of each compound in an IT–TOF mass spectrometer. Mass error for obtained masses were lower than 10 ppm.

To test the capability of the IT–TOF MS setup to detect adenosine, we directly injected solutions of both adenosine isotopes for primary detections and estimation of *m*/*z* values. The applied MS detection provided the signal for both isotopes of adenosine (Figure 1A,B). The presence of adenosine peaks in the obtained spectra in positive mode allowed us to estimate the values for *m*/*z*, AUP, and precision (Table 1). The calculated value for the precision of measurement points to the importance of the IS as the stabilizing factor compensating for possible fluctuations during mass spectrometric analysis. In addition, the MS signals for inosine, hypoxanthine, and xanthine, which are catabolites of adenosine, were recorded too (Figure 1C–E). Adenosine, inosine, and hypoxanthine were detected in positive mode, in contrast to xanthine, which was only possible to detect in negative mode. Molecular ion types for compounds detected in positive mode were [M + H]^+^ and [M − H]^−^ for hypoxanthine detected in negative mode. We also tested the possibility of detecting the final product of adenosine catabolism, uric acid, but while it has similar structure to other compounds of interest, the current hardware setup did not allow for its detection. The estimated *m*/*z* for ^12^C_10_,^14^N_5_ adenosine—268.1017, ^13^C_10_,^15^N_5_ adenosine—283.1225, inosine—269.0858, hypoxanthine—137.0449, and xanthine—151.0292, were within acceptable fluctuation. For all detected compounds, the estimated variation between theoretical and estimated mass error values was lower than 10 ppm.

With a focus on adenosine, we tested the possibility of its chromatographic separation on polar or ionic stationary phases. We tested three commercially available columns, SeQuant^®^ ZIC^®^-cHILIC, SeQuant^®^ ZIC^®^-pHILIC, and YMC-Triart Diol-HILIC. Most other methods developed for adenosine estimation use either reversed phase columns [22] or, when using HILIC, amide stationary phases [15]. On the other hand, our tested columns have either zwitterionic phases, which means they have positive as well as negative charge, or, as in case of YMC-Triart, contain diol groups. These phases leverage the unique properties of zwitterionic materials to enhance selectivity and retention of analytes, thus, enabling more precise and reliable quantification in complex sample matrices.

**Figure 2:**
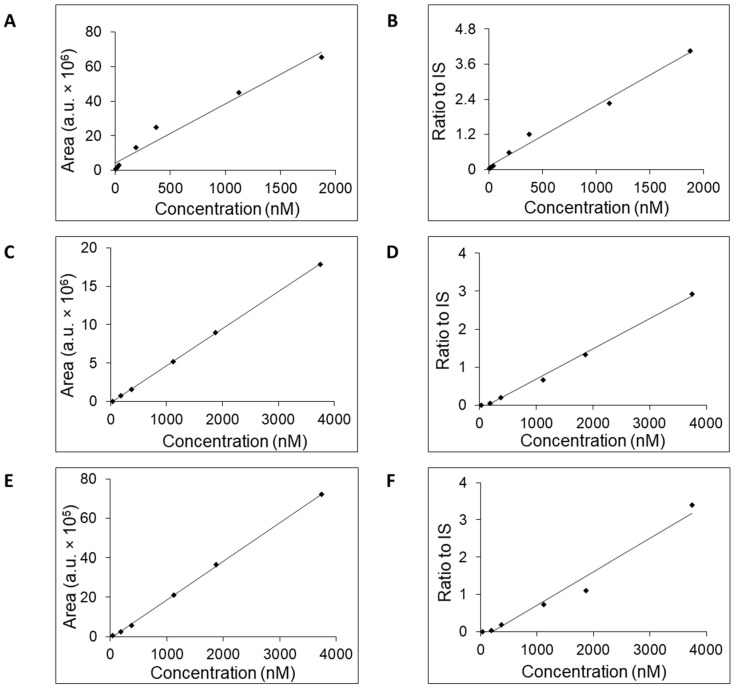
Calibration curves of adenosine obtained by LC–MS analysis either on SeQuant^®^ ZIC^®^-cHILIC (**A**,**B**), SeQuant^®^ ZIC^®^-pHILIC (**B**,**C**), or YMC-Triart Diol-HILIC (**E**,**F**) column. The adenosine concentration was plotted against its AUC values (**A**,**C**,**E**) or the calculated ratios of AUC values for adenosine to IS (**B**,**D**,**F**).

All three columns readily retained adenosine and were used to generate calibration curves (Figure 2) and to estimate the LOQ, concentration range, linearity, accuracy, and retention time (Table 2). Potential linearity was tested in mass concentration range from 0.0005 μg/mL to 10 μg/mL and working range was selected based on obtained data. Mass concentration that represents first tested point, 0.0005 μg/mL, was below established LOQ. In Table 2 are shown ranges from LOQ to the highest point where the curve was still linear. Retention time was stable for each column with small acceptable fluctuation—±0.1 min. For further work, we selected the column SeQuant^®^ ZIC^®^-cHILIC due to favorable estimated values for LOQ, accuracy, and concentration range compared to the remaining two columns. Moreover, addition of IS had a positive effect on the linearity on the SeQuant^®^ ZIC^®^-cHILIC column. The obtained values of SeQuant^®^ ZIC^®^-cHILIC performance are comparable with the HILIC–MS/MS protocol proposed by the research group of Gika [15] when a BEH Amide column was used.

**Figure 3:**
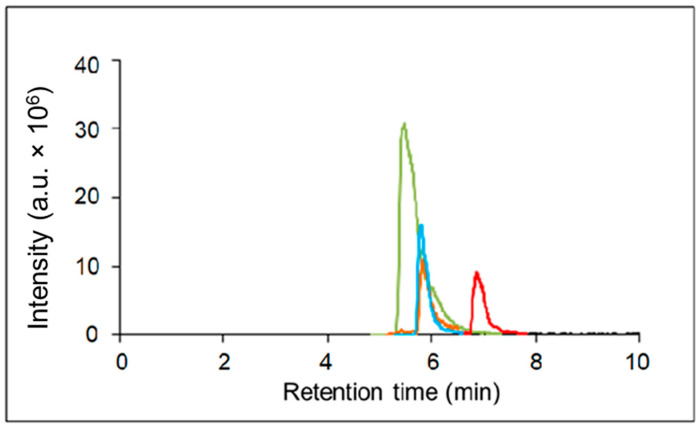
Representative chromatogram of adenosine (green), xanthine (blue), hypoxanthine (orange), and inosine (red) after their separation on SeQuant^®^ ZIC^®^-cHILIC column. The compounds were detected after elution based on their *m*/*z* values by IT–TOF MS. For representative purposes, high-mass concentrations of analytes were used; mass concentration of each analyte was 10 μg/mL.

**Figure 4:**
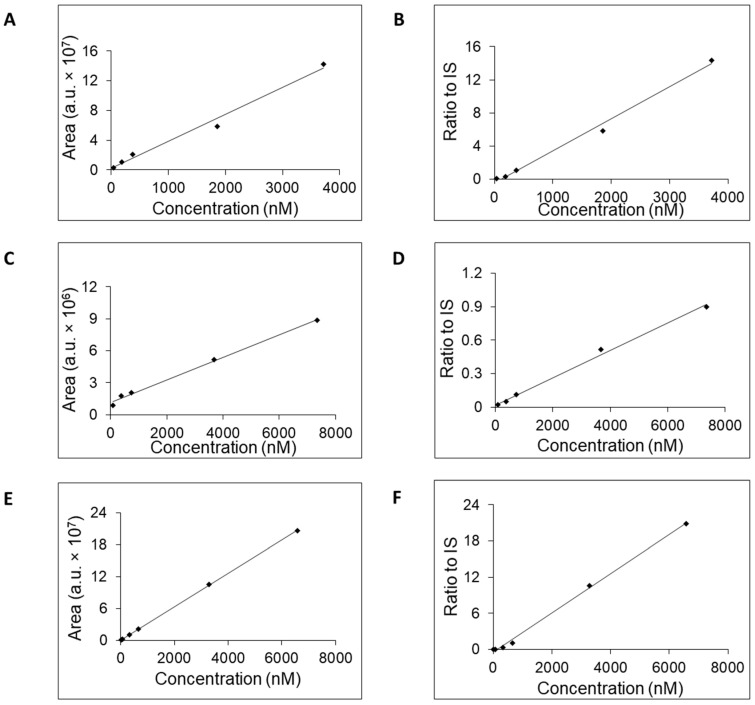
Calibration curves of inosine (**A**,**B**), hypoxanthine (**C**,**D**), and xanthine (**E**,**F**) as measured AUC values (**A**,**C**,**E**) or calculated ratio to IS (**B**,**D**,**F**).

Since the column also retained inosine, hypoxanthine, and xanthine (Figure 3, Supplementary, Figures S1–S4), the calibration curves for these three compounds were generated (Figure 4). We tested retention times, LOQ, and linear range in the same way as for adenosine. The values of the tested mass concentrations ranged from 0.0005 μg/mL to 10 μg/mL. Retention times were stable for each compound, with small acceptable fluctuation—±0.1 min. The calibration curves were generated either without or with added isotopically enriched ^13^C_10_,^15^N_5_-adenosine that served the role of the IS. The exploitation of IS addition into the samples in the process of calibration curve generation increased their linearity (Table 3) and accuracy [23,24]. Use of IS also provides benefits other than increasing linearity and accuracy. Most of its benefits come when dealing with real samples, which, when added before protein precipitation or extraction, can compensate for any sample manipulation or losses from enzymatic or any other chemical origins. IS has the same structure as adenosine, or is similar to its catabolites, which means that it acts the same as analytes. Additionally, the inclusion of IS in analytical processes enhances not only the precision but also the reliability of results, helps prevent challenges associated with sample preparation, and offers a robust framework for quantitative analysis [25].

To estimate the level of the total adenosine in blood, the freshly collected blood samples were transferred into the tubes containing ^13^C_10_,^15^N_5_-adenosine (see Section 3.2). The analysis of the processed blood samples reveals that the levels of adenosine and inosine in all tested samples are above LOQ in contrast to hypoxanthine (Table 4). The obtained MS signal for xanthine was sufficiently high in two of five samples to allow for its quantification (Table 4). The estimated values for the molar concentration of total adenosine in blood varied from 0.1 to 4.3 μM, and of inosine were from 1.15 to 8.18 μM. Those values are comparable with already published data about the adenosine and inosine content in human fluids [26,27,28]. Although the concentration of hypoxanthine in blood could reach a micromolar level [29], the applied method did not allow for it to be detected.

The usability of novel polar stationary phases for the separation of molecules prior to their detection and quantification by MS or other suitable technique provides the possibility of streamlining the analysis of biological matrices for their composition. Proposed simplification in biological sample processing includes the addition of acetonitrile to a sample already collected into a tube with IS, which is clarified by centrifugation prior to subsequent analysis. Our results provide the evidence that the proposed approach for adenosine estimation by exploiting the preloaded collection tubes with internal standard provides a novel approach for LC–MS-based quantification of compounds with high precision, sensitivity, and accuracy. The comparability of our estimated results with the suggested method demonstrates a less time-consuming procedure of liquid sample handling that can lead to LC–MS data, which are comparable in terms of sensitivity and accuracy with previously published protocols [15,22,30]. Additional benefit of our proposed protocol is the possibility of parallel estimation of adenosine catabolites, i.e., inosine, hypoxanthine, and xanthine. The simplicity of our protocol might be well-exploitable in biomedical research, diagnostics, or medical praxis.

## 3. Materials and Methods

Blood samples of five randomly selected patients with repeat syncopal episodes were selected for evaluation of the method. The blood was collected and examined for blood adenosine levels during the clinical differential diagnostic process. Venous blood was taken from antecubital vein. Blood samples were processed as reported in sample processing section. All patients agreed with study participation, and signed a written informed consent for study participation prior to blood sampling.

### 3.1. Standards

Adenosine, inosine, xanthine, hypoxanthine, and ^13^C_10_,^15^N_5_-adenosine were purchased in highest available purity from Sigma Aldrich, Germany. Uric acid was purchased from Erba Lachema. All standards were available in solid form. Standards were weighted and dissolved in water in order to prepare stock solutions. Mass concentration of all stock solutions of compounds of interest was 1 g/L. The stock solution of ^13^C_10_,^15^N_5_-adenosine was mixed with acetonitrile (Honeywell, Germany) to prepare acetonitrile enriched with ^13^C_10_,^15^N_5_-adenosine to the level of 0.1 mg/L. In this case, ^13^C_10_,^15^N_5_-adenosine served the role of internal standard (IS). All other stock standard solutions were diluted to a mass concentrations 10 times higher than working concentration with water, whereupon standard solutions were diluted to final working mass concentrations with acetonitrile containing IS, for solutions to contain 90% (*v*/*v*) acetonitrile, water. For all experiments, ultra-pure water generated by Milli-Q instrument was used, the resistivity of water was at least 18.2 MΩ·cm at 25 °C; with total organic carbon ≤ 5 ppb.

### 3.2. Collection Tubes Preparation

Collection tubes are plastic tubes preloaded with IS. Acetonitrile containing IS is added to the tubes, solvent is evaporated, and tubes are enriched with isotopically labeled adenosine. The procedure was performed as stated. From solution containing 10 μg/mL of IS in acetonitrile, 100 μL was added into plastic collection tubes (BioRad, Hercules, CA, USA). The tubes were weighed before and after the addition of solution with internal standard. Mass difference of empty tubes and tubes with IS solution served for calculation of exact volume of adenosine solution in acetonitrile with the aim of estimating the amount of adenosine that remained in tube after subsequent evaporation in Concentrator plus (Eppendorf, Hamburg, Germany). For this calculation, the used value of density for acetonitrile was 786 kg/m^3^. For calculation of adenosine in tubes, firstly the exact volume of acetonitrile was calculated by multiplication of mass difference of empty tubes and tubes with IS solution by acetonitrile density. Then, this exact volume was multiplied by mass concentration of IS in stock solution for each tube.

### 3.3. Sample Processing

After collection, 0.5 mL of blood was immediately transferred to prepared tubes containing 0.1 μg of IS, mixed with 1.5 mL of acetonitrile, and the mixture was vortexed. Samples were then stored at −20 °C. Before analysis, samples were vortexed and centrifuged at 10,000× *g* for 10 min. Subsequently, 100 μL of supernatant was diluted with 400 μL of acetonitrile, frozen for 30 min at −20 °C for protein precipitation, followed by centrifugation at 10,000× *g* for 10 min. Supernatants were collected and transferred to glass vials for LC–MS analysis. 

### 3.4. Liquid Chromatography–Mass Spectrometry Analysis

Prepared samples were analyzed on LC–ESI–IT–TOF setup (Shimadzu, Kyoto, Japan) consisting of Liquid Chromatograph Nexera X2 and IT-TOF^TM^ mass spectrometer. Parts for liquid chromatography include degasser (DGU-20A3R), two high pressure pumps (LC-30AD), autosampler (SIL-30AC), thermostat (CTO-20AC), and communication module (CBM-20A). For chromatographic separation of adenosine, three commercially available columns were tested, i.e., SeQuant^®^ ZIC^®^-cHILIC (3 μm, 100 A, 150 × 2.1 mm) (Sigma Aldrich, Steinheim, Germany), SeQuant^®^ ZIC^®^-pHILIC (5 μm, 150 × 2.1 mm) (Sigma Aldrich, Steinheim, Germany), and YMC-Triart-Diol-HILIC (1.9 μm, 150 × 2.1 mm) (YMC, Dinslaken, Germany). Manufacturer recommendations were considered at initial conditions for usage of columns. The mobile phase composition as well as applied gradients were based on manufacturer references, in order to prolong lifespan of columns as much as possible. Concentration of salt in mobile phase was kept as low as possible to also protect components of mass spectrometer. Separation on SeQuant^®^ ZIC^®^-cHILIC column gradient was similar to our previous work, but was slightly changed to manufacturer recommendation in order to extend its lifespan [31].

Two μL of sample was injected to LC–MS via autosampler at 4 °C. For chromatography separation on SeQuant^®^ ZIC^®^-cHILIC column, the column was equilibrated with initial composition of mobile phases for gradient elution. Applied gradient was as follows: mobile phase A consisted of 20 mM ammonium formate (Sigma Aldrich, Steinheim, Germany) solution in water and B of 100% acetonitrile (Honeywell, Charlotte, NC, USA). Initial conditions were 10% A at a total flow rate of 0.2 mL/min. Subsequently, gradient changed to 80% A in 24 min. After that, A returned to 10% in 6 min and an additional 2 min was used to re-equilibrate the column before next injection.

Chromatographic separation on SeQuant^®^ ZIC^®^-pHILIC was performed by following gradient application. Initial conditions were 10% of phase A and 90% B. Gradient changed to 80% A in 24 min. Subsequently, mobile phase composition returned to 10% in 6 min, and additional 2 min were added for column equilibration. Total flow was set to 0.2 mL/min.

For chromatographic separation on YMC-Triart-Diol-HILIC column, the same solutions A and B were used. The sample was injected on a column that was preequilibrated with 10% A. Subsequently, the gradient changed to 60% A in 13 min and then returned back to 10% of A in 7 min. The column was equilibrated with 10% A for next 3 min. Thermostat for chromatographic separation on all columns was set at 40 °C.

Before and after each injection, needle was rinsed with 90% acetonitrile (*v*/*v*) to eliminate any potential carry-over. Moreover, after injection of highest point during linearity testing, we injected blank multiple times to ensure that no carry-over was observed.

Data were acquired in both modes, positive and negative. Ion spray voltage was set to 4.5 kV for positive and −3.5 kV for negative mode, and ion accumulation time was 40 ms. The temperature of the heat block and capillary desolvation line were 200 °C. Nitrogen nebulizing gas flow was 1.5 L/min and drying gas pressure was set to 200 kPa. The hardware was operated by LCMS Solutions v. 3.81 (Shimadzu) software.

The chromatographic separation was omitted to test the capability of the used setup to detect the compounds of interest. In this case, direct injection mass spectrometry analysis (DIMS) was performed by injecting of two μL of standard solution with dissolved compound to mass concentration of 1 μg in one ml of solution A. Mass spectrometry conditions were same as for analysis with LC. Total flow was 0.2 mL/min and mobile phase composition was 50% A and 50% B. Analysis was performed in 2 min—analytes were detected under 30 s but some time was left to flush the capillaries. All compounds were detected as [M+H]^+^ ions but xanthine was detected as [M-H]^−^ ion. No carry-over was observed for any of tested compounds.

### 3.5. Data Analysis

Acquired spectra were directly converted to mzxml format with LCMS Solutions v. 3.81 (Shimadzu) software and imported to Skyline v. 23.1. (MacCross Lab Software), an open-source software for LC–MS data processing (https://skyline.ms (accessed on 1 December 2023)). Peaks of individual metabolites were extracted in order to obtain information such as mass-to-charge ratio (*m*/*z*), retention time, and area under the peak (AUP). We obtained AUP by manual integration of peaks, in selected time range, which was adjusted when retention time fluctuation occurred. Retention times were stable and variation was ±0.1 min. Metabolites identification was based on confirmation of retention time and *m*/*z* with standards. Mass-to-charge ratios were also confirmed with online libraries such as Pubchem (https://pubchem.ncbi.nlm.nih.gov/ (accessed on 2 September 2022)) and mzCloud (https://www.mzcloud.org/ (accessed on 2 September 2022)).

IS was used to obtain ratios for all analytes by dividing AUP of analyte with AUP of IS. Accuracy percentage was estimated by division of measured concentration and theoretical concentration for each concentration point in calibration curve. Precision is expressed as relative standard deviation of AUP or ratios by repeating measurement of the same sample. Limit of quantification is the lowest concentration point in calibration curve. Linearity of the method was estimated by construction of calibration curves and calculation of coefficient of determination (R^2^).

## 4. Conclusions

We proposed and tested the method to quantify the levels of adenosine together with inosine, hypoxanthine, and xanthine in blood by LC–MS analysis. This method is based on the enrichment of a tested sample with ^13^C_10_,^15^N_5_-adenosine, which serves the role of internal standard, and retention of adenosine on the polar stationary phase before MS detection. Furthermore, possibility to pretreat the collection tubes with pre-loaded, lyophilized ^13^C_10_,^15^N_5_-adenosine offers an easy practical way to compensate for putative variation in adenosine levels in samples during handling. The employed LC–MS procedure offers high sensitivity for detecting adenosine and its catabolites in blood even at low concentrations, which is crucial for studying its regulatory roles in different physiological processes. The integration of LC–MS into adenosine research not only refines the ability to quantify this nucleoside and its catabolites in blood accurately, but also opens avenues for exploring its associations with cardiovascular dynamics, paving the way for a deeper understanding of its physiological significance and potential implications for cardiovascular health.

## Figures and Tables

**Table 1:** Estimated values of *m*/*z*, average values of area under the peak (AUP) for both isotopes of adenosine, and precision of measurement. Two ng of each adenosine isotope were directly injected into the IT–TOF mass spectrometer. Each isotope was injected six times (*n* = 6).

Compound	*m*/*z*	AUP	Precision
		10^6^ a.u.	%
^12^C_10_,^14^N_5_-Adenosine	268.1016 ± 0.0004	31.3 ± 5.4	17.4
^13^C_10_,^15^N_5_-Adenosine	283.1212 ± 0.0008	30.5 ± 5.5	18.0
Ratio	-	1.10 ± 0.01	0.6

**Table 2:** Acquired parameters for adenosine separation on three tested HILIC columns. The concentration ranges and parameters of linearity for calibration curves either for solutions of adenosine either without or enriched with internal standard (IS).

Column	Retention Time	Concentration Range nM	Linearity (R^2^)		LOQ	Accuracy
	min		w/o IS	w IS ^1^	nM	%
SeQuant^®^ ZIC^®^-cHILIC	5.5	3.75–1872	0.9702	0.9882	3.75	103.9
SeQuant^®^ ZIC^®^-pHILIC	4.9	37.45–3745	0.9998	0.9955	37.45	125.3
YMC-Triart Diol-HILIC	5.5	37.45–3745	0.9996	0.9716	37.45	117.4

^1^ The isotopically labeled ^13^C_10_,^15^N_5_-adenosine was used as an internal standard (IS) with the constant mass concentration of 90 ng/mL.

**Table 3:** The values of retention times, *m*/*z*, and linearities for inosine, hypoxanthine, xanthine, and adenosine estimated by LC–MS analysis, while separation was performed on SeQuant^®^ ZIC^®^-cHILIC column.

Compound	Retention Time	Concentration Range	Linearity (R^2^)		LOQ
	min	nM	w/o IS	w IS	nM
Inosine	6.9	37.2–3718	0.9858	0.9922	37.2
Hypoxanthine	5.8	73.5–7353	0.9951	0.9946	73.5
Xanthine	5.8	6.5–6579	0.9998	0.9977	6.5
Adenosine	5.5	3.75–1872	0.9702	0.9882	3.75

**Table 4:** The estimated concentrations of total adenosine, inosine, hypoxanthine, and xanthine in blood samples from synoptic patients. Each sample was analyzed twice.

Samples	[Adenosine] (μM)	[Inosine] (μM)	[Xanthine] (μM)
Sample 1	0.92	1.66	<LOQ
Sample 2	4.33	8.18	70.42
Sample 3	0.13	1.15	1.38
Sample 4	0.36	1.29	<LOQ
Sample 5	2.78	3.66	<LOQ
Average ± SD	1.7 ± 1.8	3.2 ± 3.0	ND.

LOQ—limit of detection, ND.—not determined.

## Data Availability

All data are available under the request.

## Supplementary Materials

The following supporting information can be downloaded at: https://www.mdpi.com/article/10.3390/ph17030345/s1. Supplementary material consists of Figure S1: chromatograms of adenosine, Figure S2: chromatogram of hypoxanthine, Figure S3: chromatogram of inosine and Figure S4: chromatogram of xanthine.

## References

[1] Boison D., Yegutkin G.G. (2019). Adenosine Metabolism: Emerging Concepts for Cancer Therapy. Cancer Cell.

[2] Dunwiddie T.V., Masino S.A. (2001). The Role and Regulation of Adenosine in the Central Nervous System. Annu. Rev. Neurosci..

[3] Fredholm B.B. (2007). Adenosine, an Endogenous Distress Signal, Modulates Tissue Damage and Repair. Cell Death Differ..

[4] Garcia-Gil M., Camici M., Allegrini S., Pesi R., Tozzi M.G. (2021). Metabolic Aspects of Adenosine Functions in the Brain. Front. Pharmacol..

[5] Guieu R., Deharo J.-C., Maille B., Crotti L., Torresani E., Brignole M., Parati G. (2020). Adenosine and the Cardiovascular System: The Good and the Bad. JCM.

[6] Haskó G., Antonioli L., Cronstein B.N. (2018). Adenosine Metabolism, Immunity and Joint Health. Biochem. Pharmacol..

[7] Ohta A. (2016). A Metabolic Immune Checkpoint: Adenosine in Tumor Microenvironment. Front. Immunol..

[8] Yegutkin G.G., Boison D. (2022). ATP and Adenosine Metabolism in Cancer: Exploitation for Therapeutic Gain. Pharmacol. Rev..

[9] Headrick J.P., Ashton K.J., Rose’Meyer R.B., Peart J.N. (2013). Cardiovascular Adenosine Receptors: Expression, Actions and Interactions. Pharmacol. Ther..

[10] Gaudry M., Vairo D., Marlinge M., Gaubert M., Guiol C., Mottola G., Gariboldi V., Deharo P., Sadrin S., Maixent J.M. (2020). Adenosine and Its Receptors: An Expected Tool for the Diagnosis and Treatment of Coronary Artery and Ischemic Heart Diseases. Int. J. Mol. Sci..

[11] Guieu R., Deharo J.-C., Ruf J., Mottola G., Kipson N., Bruzzese L., Gerolami V., Franceschi F., Ungar A., Tomaino M. (2015). Adenosine and Clinical Forms of Neurally-Mediated Syncope. J. Am. Coll. Cardiol..

[12] Altstidl J.M., Gaede L., Troebs M., Marwan M., Achenbach S. (2022). Side Effects and Major Adverse Cardiac Events Caused by Fractional Flow Reserve Measurement: A Systematic Review and Meta-Analysis of 12,215 Patients. Eur. Heart J..

[13] Borea P.A., Gessi S., Merighi S., Vincenzi F., Varani K. (2017). Pathological Overproduction: The Bad Side of Adenosine. Br. J. Pharmacol..

[14] Holčapek M., Byrdwell W.C. (2017). Handbook of Advanced Chromatography—Mass Spectrometry Techniques.

[15] Virgiliou C., Fragakis N., Sotiriadou M., Vassilikos V., Gerou S., Theodoridis G., Gika H. (2021). HILIC-MS/MS Analysis of Adenosine in Patient Blood. Separations.

[16] González-Domínguez R., Jáuregui O., Queipo-Ortuño M.I., Andrés-Lacueva C. (2020). Characterization of the Human Exposome by a Comprehensive and Quantitative Large-Scale Multianalyte Metabolomics Platform. Anal. Chem..

[17] Capogrossi M.C., Holdiness M.R., Israili Z.H. (1982). Determination of Adenosine in Normal Human Plasma and Serum by High-Performance Liquid Chromatography. J. Chromatogr. B Biomed. Sci. Appl..

[18] Ontyd J., Schrader J. (1984). Measurement of Adenosine, Inosine, and Hypoxanthine in Human Plasma. J. Chromatogr. B Biomed. Sci. Appl..

[19] Thane Eells J., Spector R. (1983). Purine and Pyrimidine Base and Nucleoside Concentrations in Human Cerebrospinal Fluid and Plasma. Neurochem. Res..

[20] Kaufmann I., Hoelzl A., Schliephake F., Hummel T., Chouker A., Lysenko L., Peter K., Thiel M. (2007). Effects of Adenosine on Functions of Polymorphonuclear Leukocytes from Patients with Septic Shock. Shock.

[21] Löfgren L., Pehrsson S., Hägglund G., Tjellström H., Nylander S. (2018). Accurate Measurement of Endogenous Adenosine in Human Blood. PLoS ONE.

[22] Doležalová P., Krijt J., Chládek J., Němcová D., Hoza J. (2005). Adenosine and Methotrexate Polyglutamate Concentrations in Patients with Juvenile Arthritis. Rheumatology.

[23] Tan A., Boudreau N., Lévesque A., Xu Q.A., Madden T.L. (2012). Internal Standards for Quantitative LC-MS Bioanalysis. LC-MS in Drug Bioanalysis.

[24] Bergeron A., Furtado M., Garofolo F. (2009). Importance of Using Highly Pure Internal Standards for Successful Liquid Chromatography/Tandem Mass Spectrometric Bioanalytical Assays. Rapid Commun. Mass Spectrom..

[25] Pitt J.J. (2009). Principles and Applications of Liquid Chromatography-Mass Spectrometry in Clinical Biochemistry. Clin. Biochem. Rev..

[26] Traut T.W. (1994). Physiological Concentrations of Purines and Pyrimidines. Mol. Cell Biochem..

[27] Human Metabolome Database: Showing Metabocard for Adenosine (HMDB0000050). https://hmdb.ca/metabolites/HMDB0000050.

[28] Human Metabolome Database: Showing Metabocard for Inosine (HMDB0000195). https://hmdb.ca/metabolites/HMDB0000195.

[29] Human Metabolome Database: Showing Metabocard for Hypoxanthine (HMDB0000157). https://hmdb.ca/metabolites/HMDB0000157.

[30] Hui Y., Zhao S.S., Love J.A., Ansley D.M., Chen D.D.Y. (2012). Development and Application of a LC–MS/MS Method to Quantify Basal Adenosine Concentration in Human Plasma from Patients Undergoing on-Pump CABG Surgery. J. Chromatogr. B.

[31] Gondáš E., Kráľová Trančíková A., Baranovičová E., Šofranko J., Hatok J., Kowtharapu B.S., Galanda T., Dobrota D., Kubatka P., Busselberg D. (2022). Expression of 3-Methylcrotonyl-CoA Carboxylase in Brain Tumors and Capability to Catabolize Leucine by Human Neural Cancer Cells. Cancers.

