# Supplementary material for: Application of Liquid Chromatography Coupled to Mass Spectrometry for Direct Estimation of the Total Levels of Adenosine and Its Catabolites in Human Blood
Source: Pharmaceuticals (Basel). 2024 Mar 7;17(3):345. doi: 10.3390/ph17030345 (PMC10974345; doi:10.3390/ph17030345)
Supplement: Supplementary file 1 [file pharmaceuticals-17-00345-s001.zip › pharmaceuticals-2883564-supplementary.pdf]

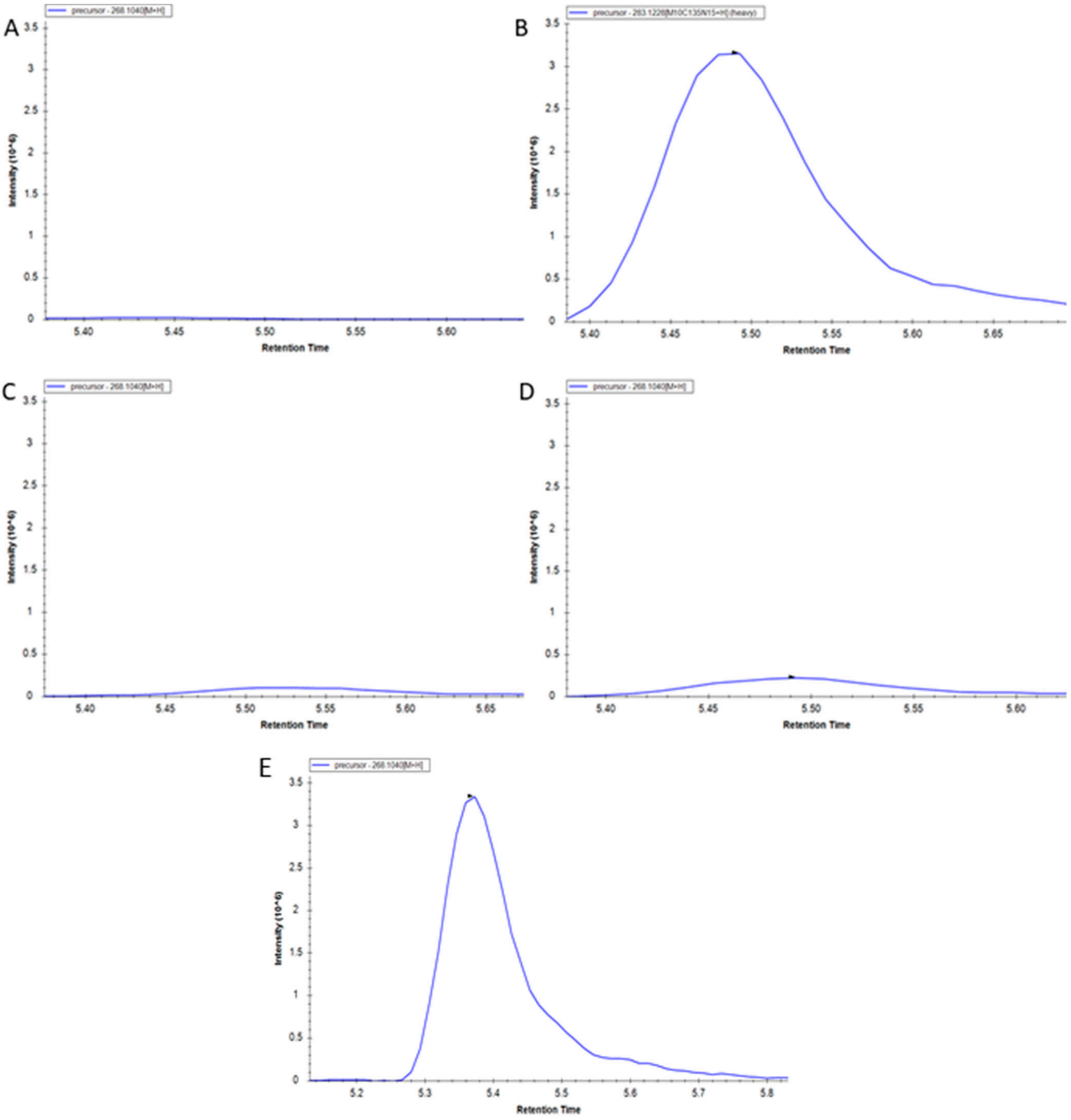

Figure S1. Representative chromatograms of a mobile phase (**A**), internal standard (**B**), adenosine at LOQ (**C**), which is 3.75 nM, blood sample (**D**) and adenosine at HOQ (**E**), which is 1872 nM.

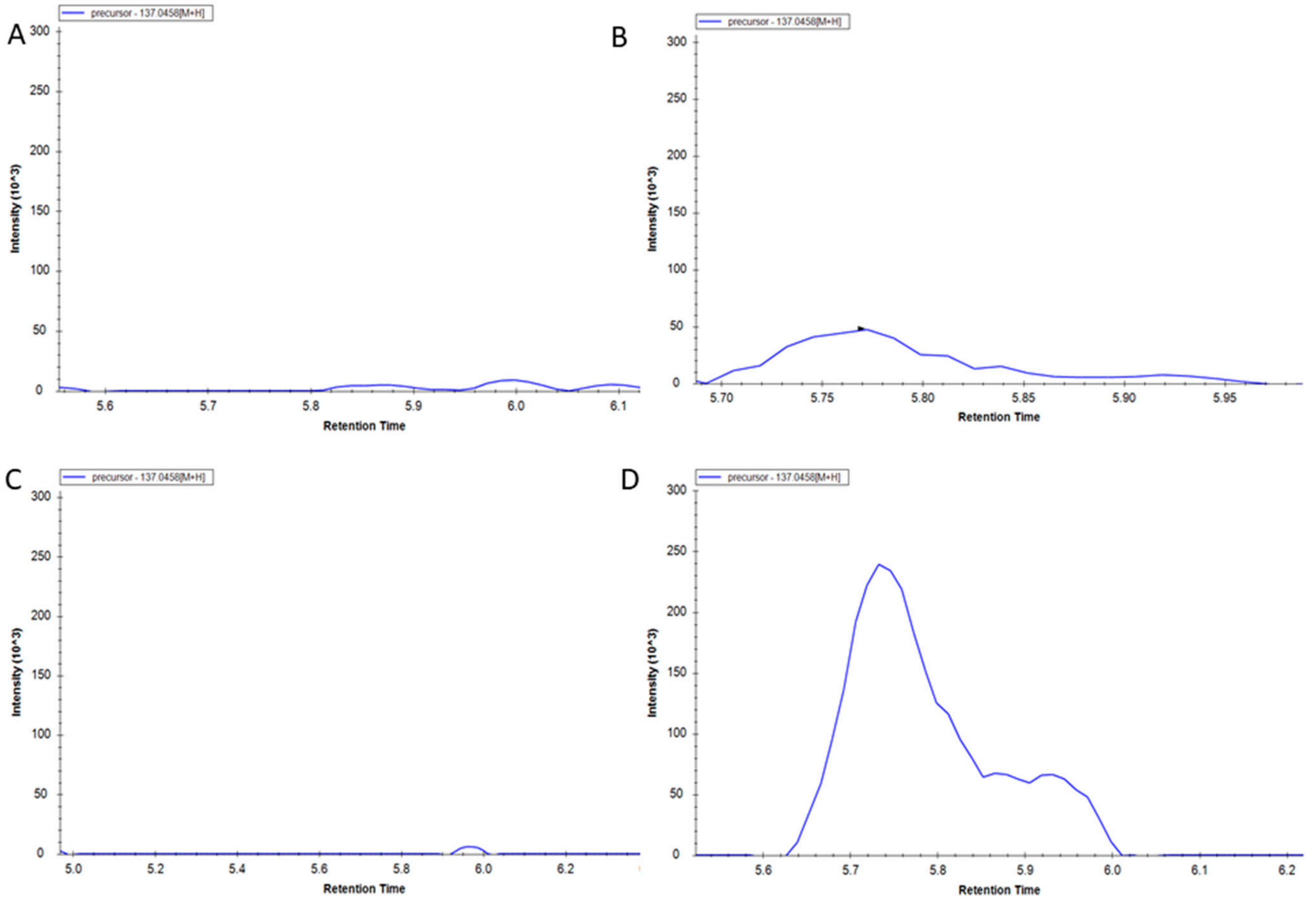

Figure S2. Representative chromatograms of a mobile phase (**A**), hypoxanthine at LOQ (**B**), which is 73.5 nM, blood sample (**C**) and hypoxanthine at HOQ (**D**), which is 7353 nM.

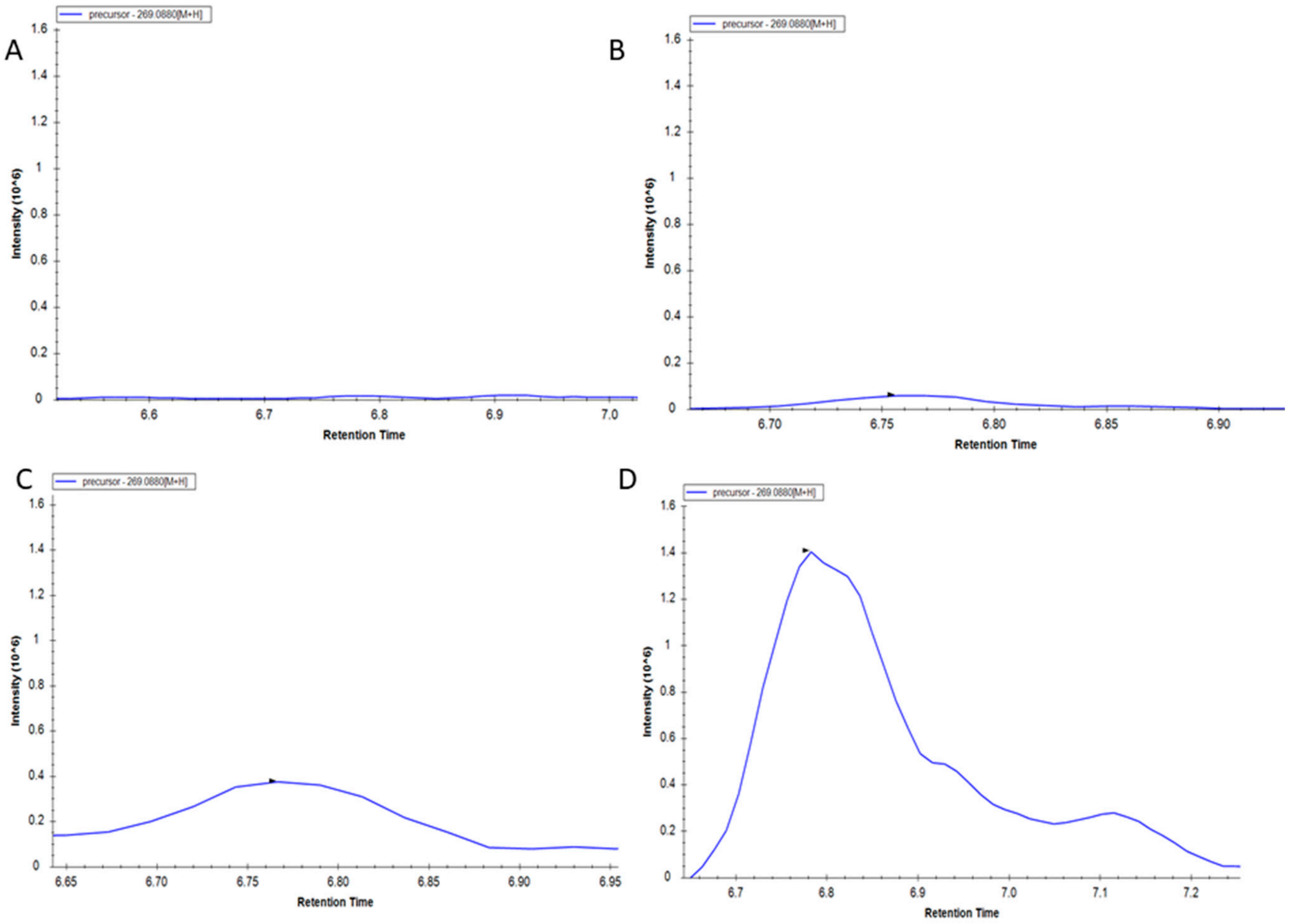

Figure S3. Representative chromatograms of a mobile phase (**A**), inosine at LOQ (**B**), which is 37.2 nM, blood sample (**C**) and inosine at HOQ (**D**), which is 3718 nM.

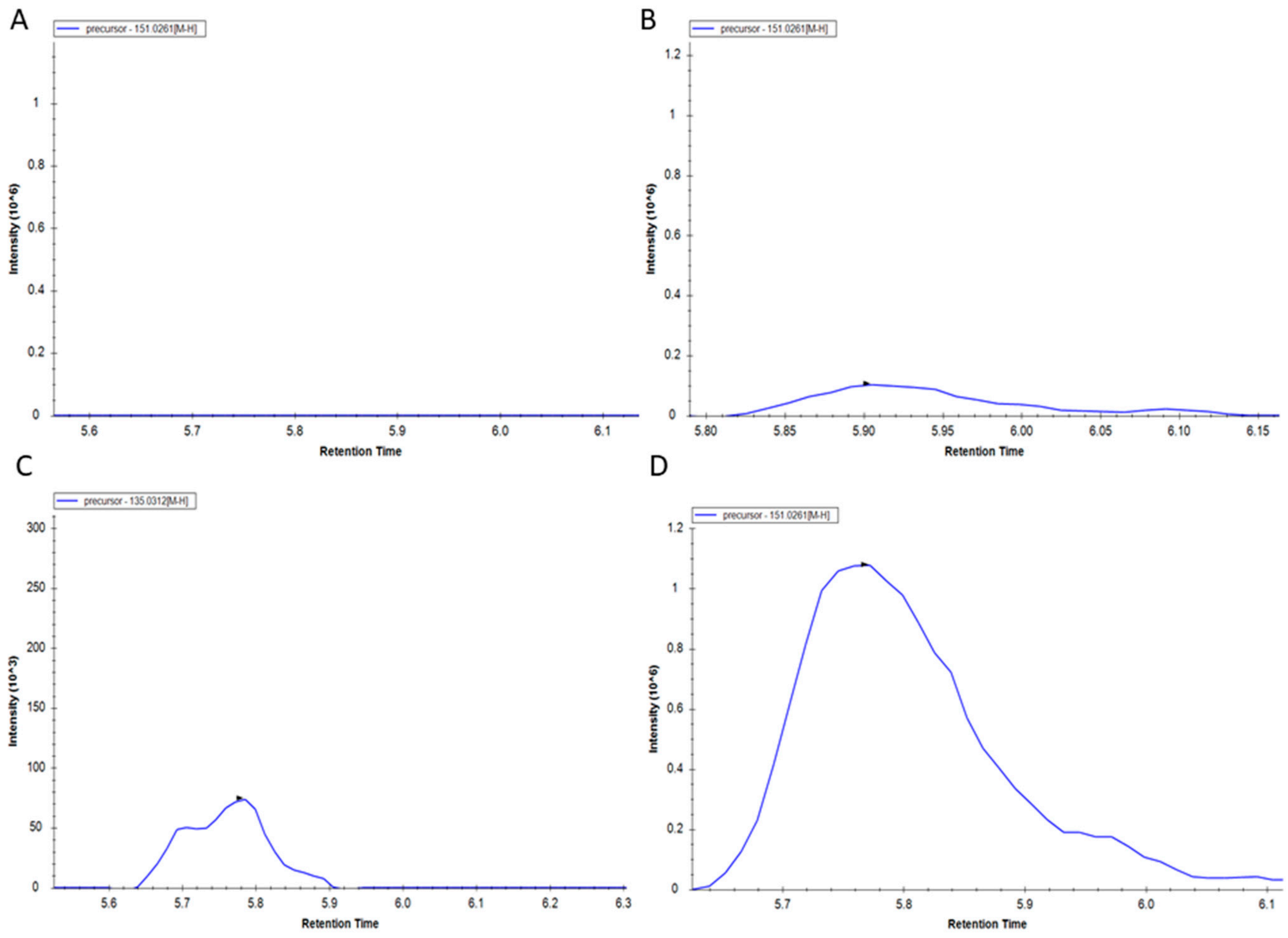

Figure S4. Representative chromatograms of a mobile phase (**A**), xanthine at LOQ (**B**), which is 6.5 nM, blood sample (**C**) and xanthine at HOQ (**D**), which is 6579 nM.
